# Deciphering the coagulation profile through the dynamics of thrombin activity

**DOI:** 10.1038/s41598-020-69415-y

**Published:** 2020-07-27

**Authors:** Romy M. W. de Laat-Kremers, Qiuting Yan, Marisa Ninivaggi, Moniek de Maat, Bas de Laat

**Affiliations:** 1 Synapse Research Institute, Pastoor Habetsstraat 50, 6217 KM Maastricht, The Netherlands; 2 Department of Biochemistry, CARIM, Maastricht University, Maastricht, The Netherlands; 3 Department of Hematology, Erasmus University Medical Center, Rotterdam, The Netherlands

**Keywords:** Biochemistry, Biomarkers, Experimental models of disease, Preclinical research, Translational research

## Abstract

Thrombosis has proven to be extremely difficult to predict. Measuring the generation of thrombin is a very sensitive method to detect changes in the hemostatic system. We developed a method based on the generation of thrombin to further fingerprint hemostasis, which we have named thrombin dynamics. Via this method we are able to exactly measure the prothrombin conversion and thrombin inactivation, and any change in the coagulation cascade will be reflected in these two processes. In the current study we analyzed the importance of the members of the prothrombin complex on the dynamics of thrombin activation and inactivation. We show that prothrombin conversion is predominantly influenced by factor X and antithrombin, which will provide essential insights in complex thrombosis-related diseases, such as liver cirrhosis and kidney failure.

## Introduction

Thrombin is the key enzyme in the coagulation cascade and converts fibrinogen into a fibrin network. The thrombin generation (TG) test measures the amount of thrombin that is generated in plasma in response to a tissue factor stimulus^1^. TG is a widely used method to screen for hyper- and hypo-coagulability^2^, as increased TG is associated with thrombosis, and vice versa, reduced TG is related to bleeding^2–8^. Additionally it is often used to assess therapeutic strategies, both in research^9,10^ and in the clinic^11,12^. It is a global coagulation assay and subsequently, a deviant TG profile cannot be immediately attributed to a specific coagulation defect^1,2^ and further testing is required.

**Figure 1:**
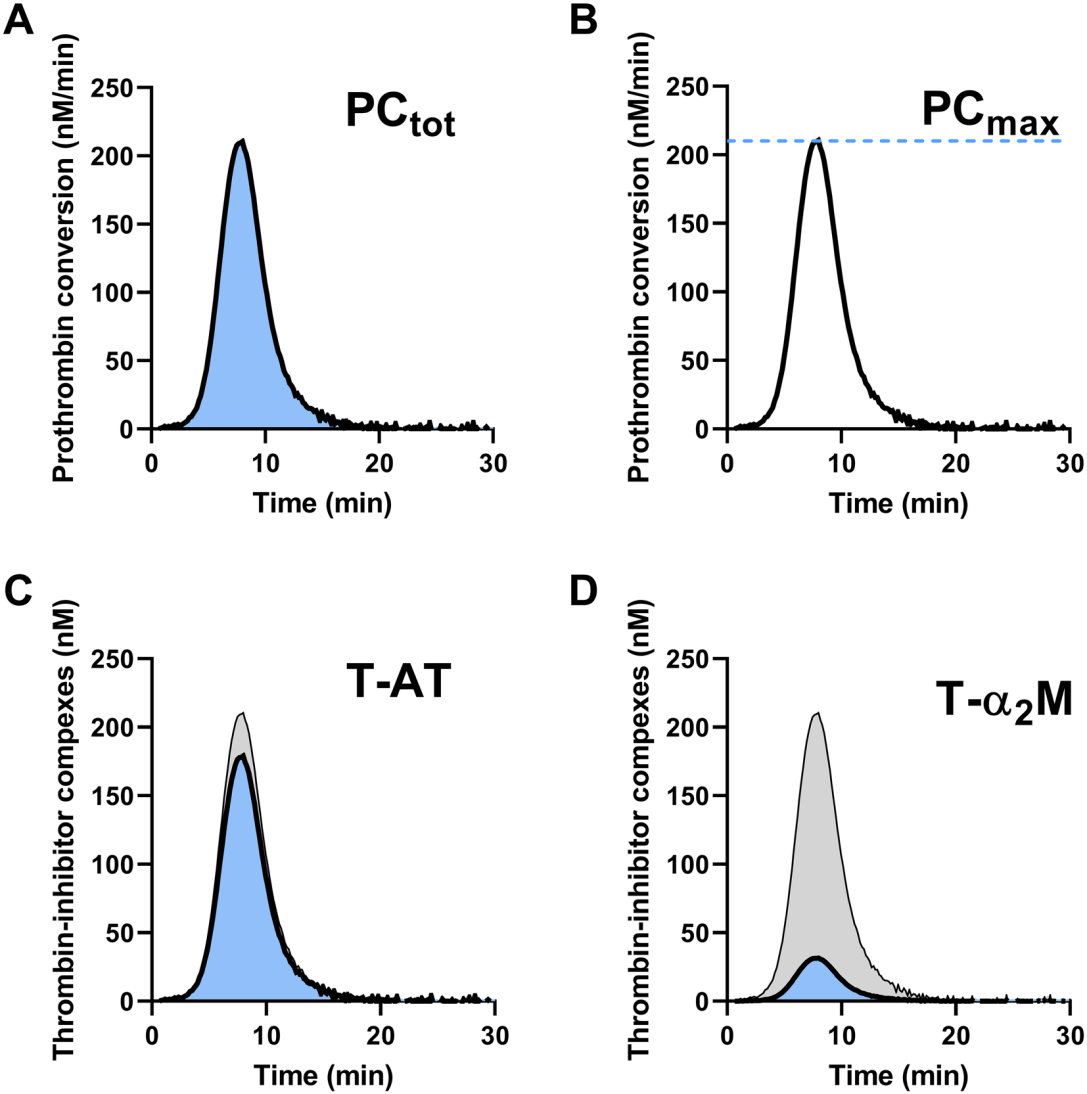
Illustration of the quantification of thrombin dynamics parameters. (**A**) The total amount of prothrombin converted (PC_tot_) is quantified as the area under the curve of the prothrombin conversion curve. (**B**) The maximum rate of prothrombin conversion (PC_max_) is defined as the peak of the prothrombin conversion curve. (**C**, **D**) The total amount of prothrombin converted during TG equals the total amount of thrombin-inhibitor complexes formed (gray area). This is split into thrombin-antithrombin complex formation (T-AT; **C**) and thrombin-α_2_Macroglobulin formation (T-α_2_M; **D**).

The thrombin generation describes the amount present in clotting plasma at each time point during the measurement. The thrombin concentration depends on two main underlying processes: the production of thrombin (prothrombin conversion) and inactivation of thrombin^13^. A reduction of TG can be caused by lower activation of the prothrombin conversion or increased thrombin inhibition. Recently, we developed a method called thrombin dynamics analysis to study the processes that underlie thrombin generation in more detail^14^. In this method, we quantify prothrombin conversion and thrombin inactivation from TG data, allowing these processes to be studied independently from each other. The rate of thrombin inactivation is predicted with an algorithm based on the plasma antithrombin (AT), α_2_Macroglobulin (α_2_M) and fibrinogen level^14^. Subsequently, the prothrombin conversion curve can be extracted from the thrombin generation curve. From this prothrombin conversion curve, the peak value and the area-under-the-curve are quantified (Fig. 1), respectively representing the maximum rate of the prothrombinase complex (PC_max_) and the total amount of prothrombin converted throughout the measurement (PC_tot_). The amount of thrombin-antithrombin (T-AT) and thrombin-α_2_Macroglobulin (T-α_2_M) complexes formed during the experiment are quantified. The thrombin inactivation capacity (TDC) is calculated independent from the TG curve and depends solely on the AT, α_2_M and fibrinogen level of a plasma sample.

The dynamics of thrombin generation have been studied in multiple clinical settings over the past years to study the balance between pro-and anticoagulant mechanisms, and to perform in silico experimentation to generate hypotheses^14–22^. Recently, questions have started to emerge about the influence of individual coagulation factor levels on the parameters of prothrombin conversion and thrombin inactivation. It is of interest to study the contribution of specific coagulation factors to the individual parameters in order to further fingerprint coagulation. This allows the better interpretation of in silico results and the generation of new working hypotheses based on the in silico work. Another important question that needs to be addressed is when the novel parameters should be considered abnormal. Until now, reference ranges were not available for prothrombin conversion and thrombin inactivation parameters, which makes it difficult to interpret the assays results clinically when a study is performed in a clinical setting. Especially in the case of in silico experimentation on clinical data, reference values are an important tool to define what is considered normal and what is not.

Thrombin is the last enzyme of the coagulation cascade which converts fibrinogen into fibrin thereby changing the liquid blood into a solid compound. Any change in the coagulation factors will have an effect on the generation and activity of thrombin. In the current study, we investigated the effect of individual coagulation factors mostly belonging to the prothrombinase complex on the dynamics of thrombin activity using thrombin generation, prothrombin conversion and thrombin inactivation as read out.

## Results

Even though the thrombin dynamics method has shown its added value to thrombin generation data in several clinical studies in the past years, the meaning of each parameter in the wider context of coagulation has yet to be investigated^14–22^. In this study, we investigated the effect of four major coagulation factors (prothrombin, FV, FX, and antithrombin) on five thrombin dynamics parameters (PC_tot_, PC_max_, T-AT, T-α_2_M, and TDC). We chose to study prothrombin, FV and FX because they are components of the prothrombinase complex, converting prothrombin into active thrombin. In addition, antithrombin was studied because it is the most important natural thrombin inhibitor. The role of each individual coagulation factor was investigated by performing dose–response measurements in plasma deficient in that specific factor and by performing correlation analysis in a group of healthy subjects (n = 122).

### The effect of plasma prothrombin level on the dynamics of thrombin generation

We first investigated the effect of prothrombin on thrombin generation, prothrombin conversion and thrombin inactivation. The plasma prothrombin level has a strong dose-dependent effect on thrombin generation (Supplementary Fig. 1).

**Figure 2:**
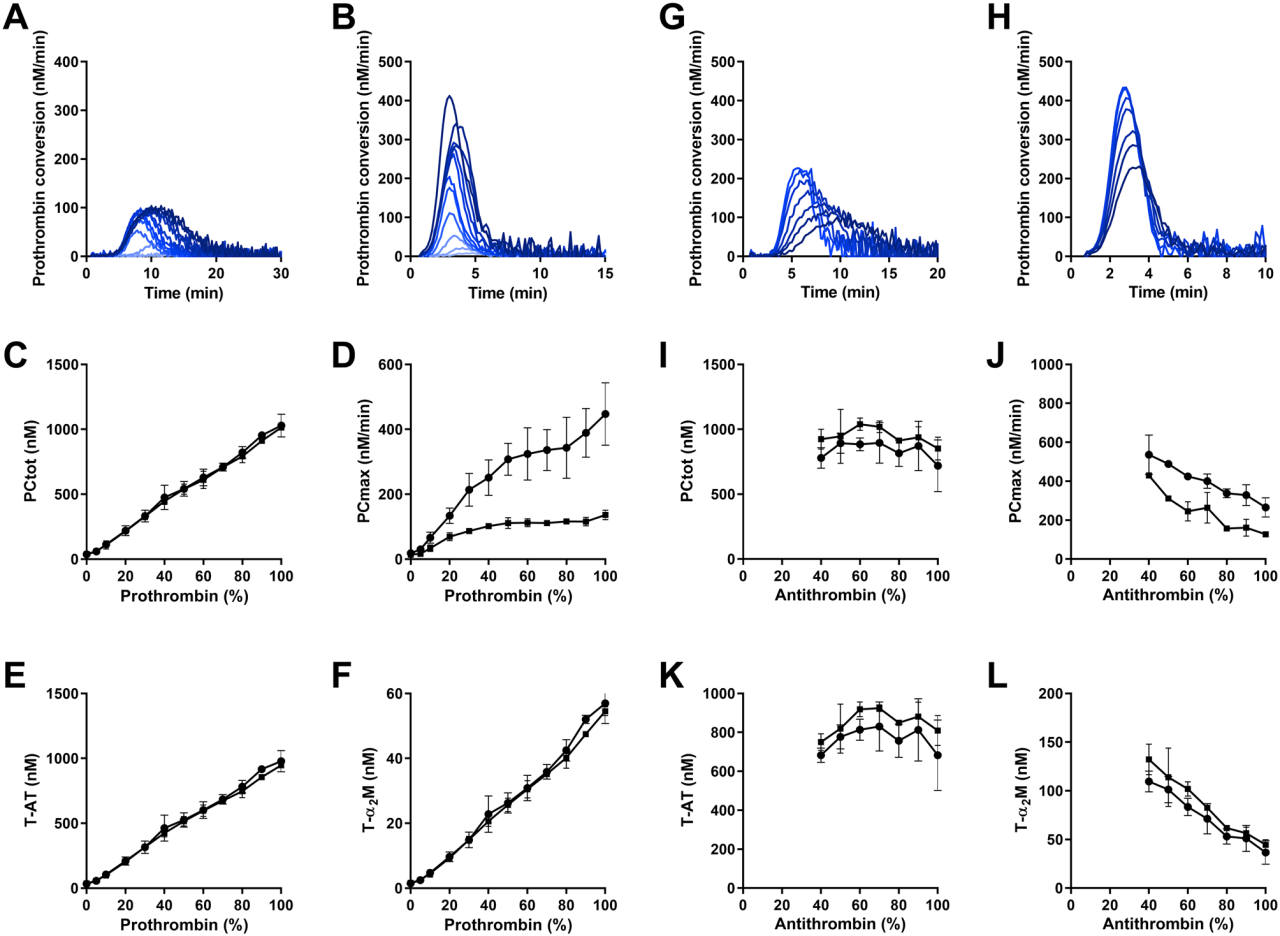
The effect of prothrombin and antithrombin on the dynamics of thrombin generation. (**A**–**F**) Prothrombin deficient plasma was mixed with pooled normal to achieve plasma concentrations of 0, 5, 10, 20, 30, 40, 50, 60, 70, 80, 90, and 100% prothrombin. Prothrombin conversion curves at 1 (**A**) and 5 pM TF (**B**) are shown (0–100% prothrombin from bottom to top) and thrombin dynamics parameters PC_tot_ (**C**), the PC_max_ (**D**), T-AT complexes (**E**) and T-α_2_M complexes (**F**) were quantified at 1 pM TF (■ symbols) and 5 pM TF (● symbols). **(G**–**L)** Antithrombin deficient plasma was mixed with pooled normal to achieve plasma concentrations of 40, 50, 60, 70, 80, 90, and 100% antithrombin. Prothrombin conversion curves at 1 (**G**) and 5 pM TF (**H**) are shown (0–100% antithrombin from top to bottom and thrombin dynamics parameters PC_tot_ (**I**), the PC_max_ (**J**), T-AT complexes (**K**) and T-α_2_M complexes (**L**) were quantified at 1 pM TF (■ symbols) and 5 pM TF (● symbols). The average results of 3 experiments are shown as the mean ± SD.

Below the threshold of 20%, prothrombin shortens the lag time and time-to-peak dose-dependently, and the prothrombin level correlates almost perfectly with ETP and peak at 5 pM TF (R^2^ = 0.972 and R^2^ = 0.971, respectively). We used thrombin dynamics analysis to further fingerprint the effect of the prothrombin level on thrombin generation. As expected, we found that prothrombin dose-dependently increases prothrombin conversion (Fig. 2A–F). Not only PC_tot_, but also T-AT and T-α_2_M are linearly correlated with the plasma prothrombin level (all R^2^ > 0.973) and PC_max_ increases with increasing prothrombin levels, which is most pronounced at high TF concentrations.

In healthy subjects, both PC_tot_ and T-AT are correlated to the plasma prothrombin level at 1 pM TF (R^2^ = 0.136 and R^2^ = 0.131, respectively with p < 0.0001) and at 5 pM TF (R^2^ = 0.183 and R^2^ = 0.195, respectively with p < 0.0001). TDC does not depend on prothrombin in in vitro dose–response experiments (Supplementary Fig. 2), nor in healthy subjects.

### The effect of plasma AT level on the dynamics of thrombin generation

Secondly, we quantified the effect of the natural anticoagulant antithrombin on thrombin generation, prothrombin conversion and thrombin inactivation. Thrombin generation was measured above an antithrombin level of 40% (Supplementary Fig. 1) and thrombin dynamics parameters were quantified (Fig. 2G–L). Due to experimental limitations, thrombin generation cannot be measured in plasma samples containing less antithrombin because then prothrombin levels exceed antithrombin levels, causing ongoing thrombin generation and subsequent substrate depletion. Antithrombin significantly prolongs the thrombin generation lag time and time-to-peak is inversely correlated with the ETP and peak (p < 0.001). We used thrombin dynamics analysis to investigate whether antithrombin only influences the inactivation of thrombin, or prothrombin conversion as well. Predominantly the maximum rate of prothrombin conversion (PC_max_) was attenuated by antithrombin (Fig. 2G-L). We also found that the amount of thrombin-α_2_M complexes formed during thrombin generation was reduced at increasing antithrombin concentrations (p < 0.001). In healthy subjects, we were able to confirm that the AT level was significantly correlated to the T-α_2_M level at 1 pM TF (R^2^ = 0.132 with p < 0.0001) and 5 pM TF (R^2^ = 0.158 with p < 0.0001). In addition, the thrombin decay constant strongly depends on the plasma AT level in the dose–response measurements (Supplementary Fig. 2) and in healthy subjects (R^2^ = 0.537, p < 0.0001).

### The effect of plasma FV level on the dynamics of thrombin generation

Factor V is one of the prominent members of the prothrombinase complex and we tested its role in thrombin generation, prothrombin conversion and thrombin inactivation. FV dose-dependently shortened the lag time and time-to-peak, especially below 20% FV (Supplementary Fig. 3).

**Figure 3:**
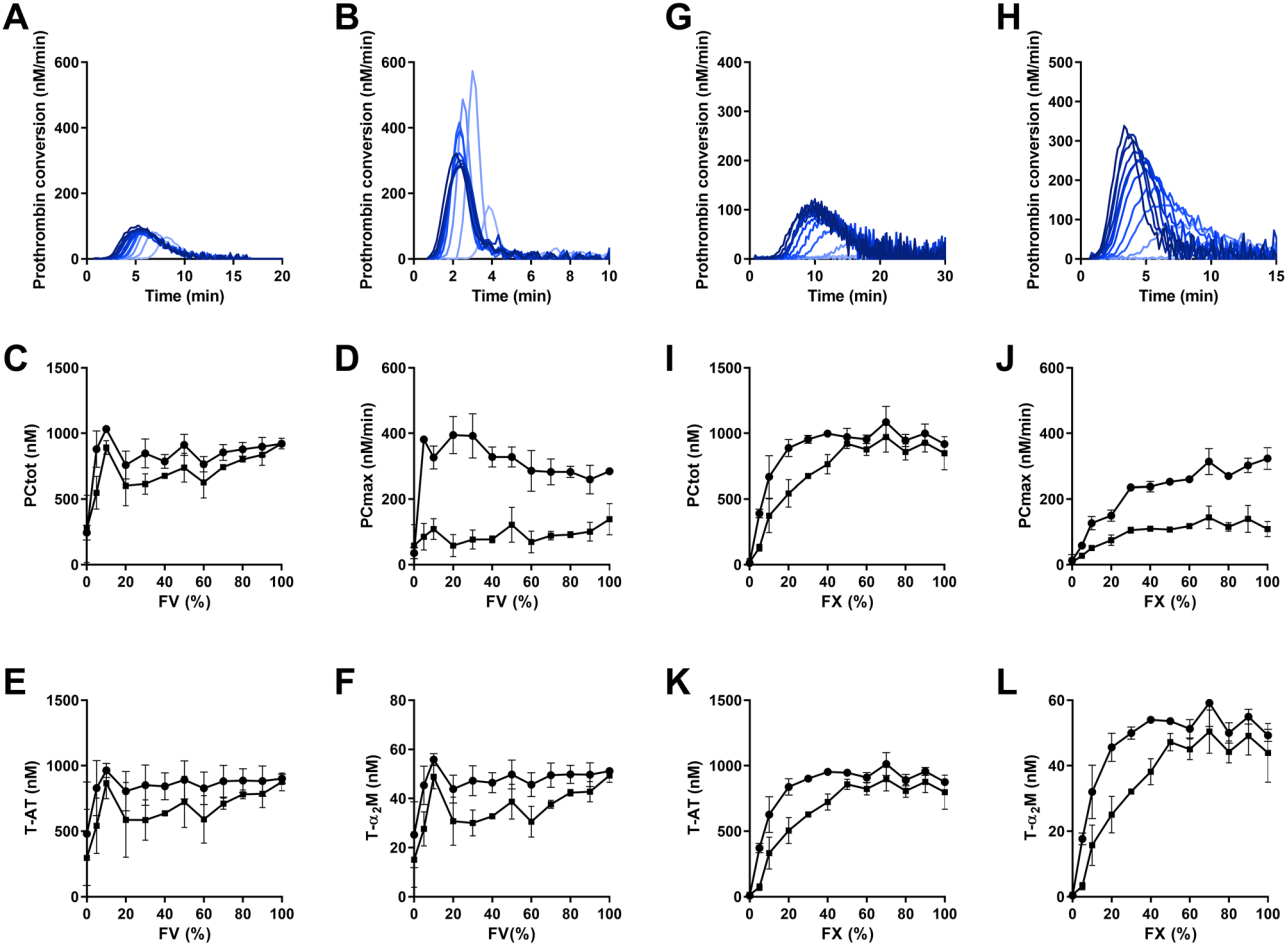
The effect of FV and FX on the dynamics of thrombin generation. (**A**–**F**) FV deficient plasma was mixed with pooled normal to achieve plasma concentrations of 0, 5, 10, 20, 30, 40, 50, 60, 70, 80, 90, and 100% FV. Prothrombin conversion curves at 1 (**A**) and 5 pM TF (**B**) are shown (0–100% FV from bottom to top) and thrombin dynamics parameters PC_tot_ (**C**), the PC_max_ (**D**), T-AT complexes (**E**) and T-α_2_M complexes (**F**) were quantified at 1 pM TF (■ symbols) and 5 pM TF (● symbols). **(G**–**L)** FX deficient plasma was mixed with pooled normal to achieve plasma concentrations of 0, 5, 10, 20, 30, 40, 50, 60, 70, 80, 90, and 100% FX. Prothrombin conversion curves at 1 (**G**) and 5 pM TF (**H**) are shown (0–100% FX from bottom to top and thrombin dynamics parameters PC_tot_ (**I**), the PC_max_ (**J**), T-AT complexes (**K**) and T-α_2_M complexes (**L**) were quantified at 1 pM TF (■ symbols) and 5 pM TF (● symbols). The average results of 3 experiments are shown as the mean ± SD.

Additionally, FV increased the TG peak height and ETP dose-dependently (p = 0.004 and p < 0.001). Using thrombin dynamics analysis, we found that the FV increases thrombin generation at low TF levels by stimulating the production of thrombin (PC_tot,_ p = 0.0023), which subsequently increases the formation of T-AT and T-a_2_M (p = 0.048 and p = 0.0045, respectively; Fig. 3A-F).

At 5 pM TF, FV did not significantly affect any of the thrombin dynamics parameters. In contrast, in healthy subjects FV did not show a significant correlation with any of the thrombin dynamics parameters. Furthermore, the thrombin decay constant did not depend on the plasma FV level (Supplementary Fig. 2), and also in the 122 healthy subjects, FV did not correlate with the thrombin decay constant.

### The effect of plasma FX level on the dynamics of thrombin generation

Factor X levels were measured and studied for their influence on thrombin. FX dose-dependently increased the thrombin generation peak height and ETP (p < 0.001 and p = 0.0004) and shortens the lag time and TTP. Thrombin dynamics analysis revealed that TG increases because of an increase of PC_tot_, T-AT and T-α_2_M in the lower range of FX (Fig. 3G–L), whereas the thrombin decay constant was not affect by the plasma FX level (Supplementary Fig. 2). Factor X levels above 40% did not increase PC_tot_, T-AT and T-α_2_M any further and resulted in a plateau (all p < 0.0001). Additionally, PC_max_ increased dose-dependently with the FX level (p < 0.0001), most pronouncedly at 5 pM TF. In healthy subjects, the plasma FX level correlates significantly with PC_tot_ at 1 and 5 pM TF (R^2^ = 0.101 and R^2^ = 0.142 with p < 0.0005) and T-AT at both 1 and 5 pM TF (R^2^ = 0.102 and R^2^ = 0.151 at p < 0.0001), but not T-α_2_M.

### Reference values for thrombin dynamics

**Figure 4:**
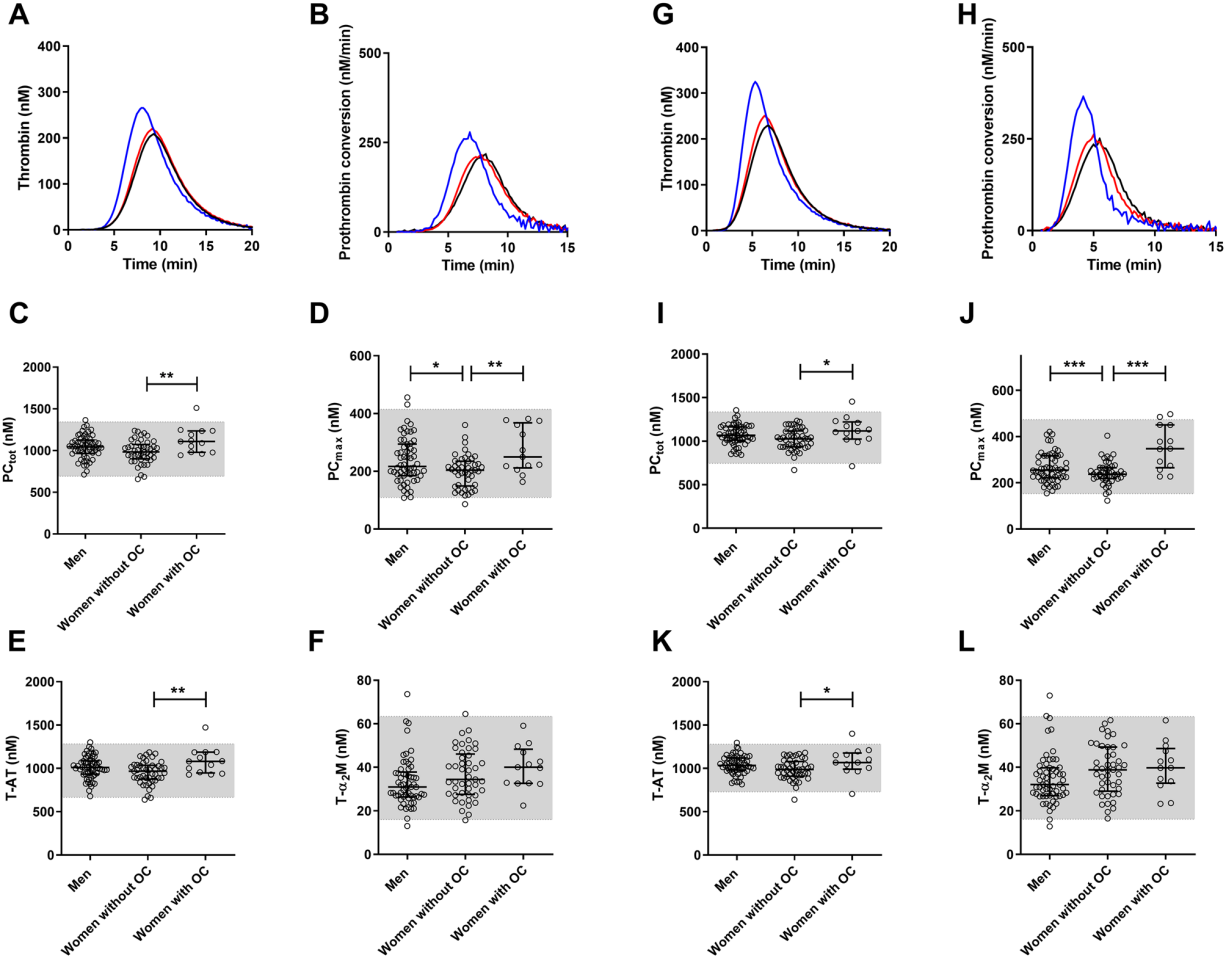
Reference values for thrombin dynamics parameters determined in 122 healthy subjects. Thrombin generation and thrombin dynamics were determined at 1 pM TF (**A**–**F**) and 5 pM TF (**G**–**L**). Average thrombin generation curves at 1 and 5 pM TF (A and G) were calculated for 3 groups: men (black), women without oral contraceptives (OC; red) and women with OC (blue). Average prothrombin conversion curve at 1 and 5 pM TF were calculated for the same groups (**B**,**H**). Reference ranges were determined for thrombin dynamics parameters at 1 and 5 pM TF: PC_tot_ (**C**,**I**), PC_max_ (**D**,**J**), T-AT (**E**,**K**) and T-α_2_M (**F**,**L**). Reference ranges are depicted as grey boxes, dots show individual values and the lines indicate the median ± interquartile range. Statistical significance was tested by ANOVA with Bonferroni correction was indicated as *p < 0.05, **p < 0.001, and *** < p < 0.001.

In order to define normal values for thrombin dynamics parameters, we measured thrombin generation and thrombin dynamics in 122 healthy subjects at 1 and 5 pM TF (Supplementary Table 1). We defined the reference ranges as the 2.5th and 97.5th percentile for the thrombin dynamics parameters in the whole group of healthy subjects (n = 122), which are depicted in Fig. 4 as grey boxes. The total amount of prothrombin converted (PC_tot_) ranged from 693 to 1,344 and from 746 to 1,335 for TG triggered with 1 and 5 pM TF, respectively. The maximum prothrombin conversion rate (PC_max_) ranged from 109 to 415 for 1 pM TF, and from 153 to 474 for 5 pM TF. Thrombin-antithrombin complexes ranged from 667 to 1,283 for 1 pM TF and from 729 to 1,279 for 5 pM and thrombin-α_2_-macroglobulin ranged from 16 to 63 both for 1 and 5 pM TF. The thrombin decay capacity ranged from 0.633 to 1.002 min^-1^ with a median value of 0.816 min^-1^.

### The dynamics of thrombin generation in men vs. women and the effect of oral contraceptives

As oral contraceptives have been shown to affect coagulation and the generation of thrombin via an effect on the protein C pathway we studied possible differences between men, women with/without OC in more detail. Figure 4 shows the average thrombin generation and prothrombin conversion curves for the subset of men (n = 60), women without oral contraceptives (OC; n = 47), and women with OC (n = 15). As expected from previous studies, thrombin generation was significantly higher in women than in men (ETP + 8%, p = 0.043) when measured at 1 pM TF. Within the group of women, the ETP was significantly higher in women using OC (+ 25%, p < 0.01) compared to women without OC. No difference was found between men and women without OC. To understand these differences in TG, we studied thrombin dynamics parameters in men, women without OC, and women with OC. We found that PC_tot_ (+ 14.4% and + 9.5% for 1 and 5 pM TF), PC_max_ (36.9 + % and + 46.1% for 1 and 5 pM TF), and T-AT (+ 14.3% and + 9.5% for 1 and 5 pM TF) were significantly elevated in women with OC compared to women without OC. T-α_2_M formation was unaffected by OC use. No differences were found between men and women without OC.

### The main determinants of thrombin dynamics parameters

**Figure 5:**
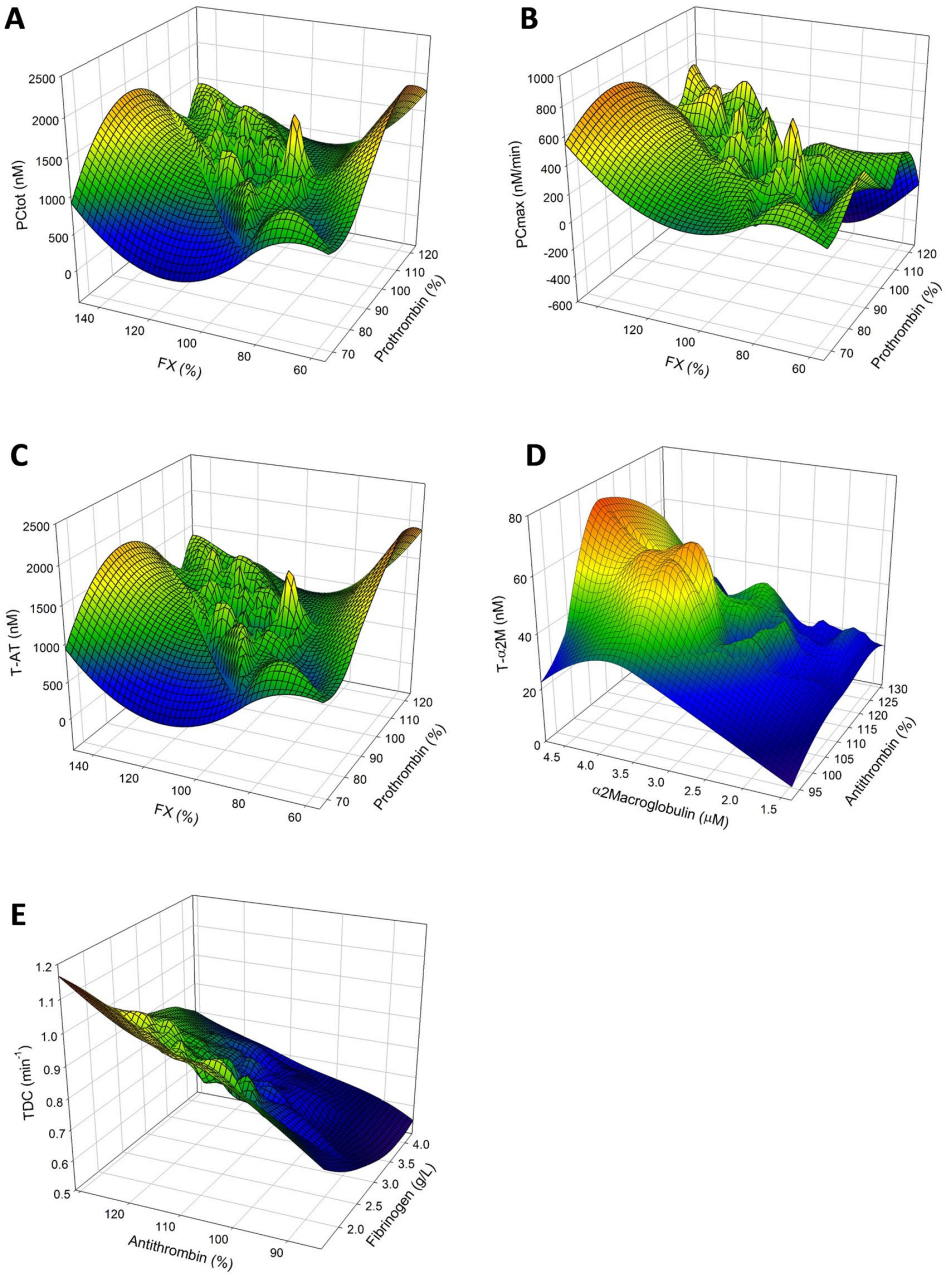
The main determinants of each thrombin dynamics parameter in 122 healthy subjects. (**A**) The influence of prothrombin and FX levels on the PC_tot_, (**B**) of prothrombin and FX levels on the PC_max_, (**C**) FII and FX levels on T-AT formation, (**D**) AT and α_2_M levels on T-α_2_M formation and (**E**) AT and fibrinogen levels on the TDC. 3D plots show the overall trend of the data as a color coded mesh, ranging from blue (low values) through green and yellow to red (high values).

We investigated in more detail possible combined effects of our input coagulation factors. Therefore, 3D plots were drawn in order to depict the relationship between each thrombin dynamics parameters and its two most important influencing coagulation factors. We found that PC_tot_ is mainly dependent on prothrombin and FX levels, and low levels of either coagulation factor lead to lower prothrombin conversion (Fig. 5A). PC_max_ is mainly dependent on prothrombin and FX (Fig. 5B). Thrombin-antithrombin formation is mostly dependent on the levels of prothrombin and FX as high prothrombin or FX levels are associated with high amounts of T-AT complexes formed (Fig. 5C). Figure 5D shows that thrombin-α_2_macroglobulin formation is high when as expected α_2_M levels are high, and T-α_2_M formation is low when α_2_M is low. The thrombin decay capacity mainly depends on antithrombin and fibrinogen levels, and high TDC is associated with high antithrombin levels and low fibrinogen levels (Fig. 5E).

### Prothrombin conversion and thrombin inactivation in haemophilia A

**Figure 6:**
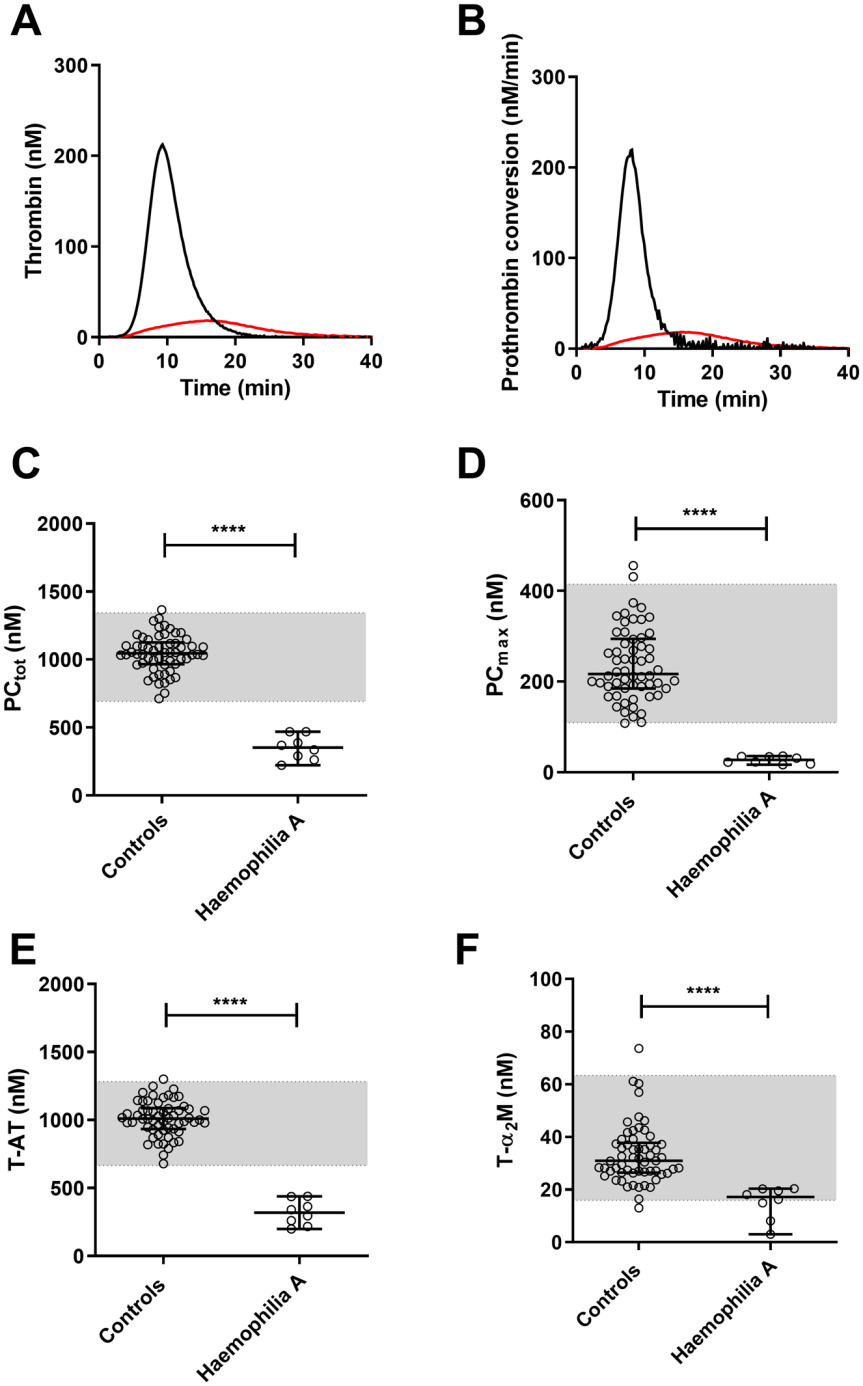
Prothrombin conversion and thrombin inactivation in haemophilia A. Thrombin generation and thrombin dynamics were determined at 1 pM TF in 60 (male) controls and 8 hemophilia A patients . Average thrombin generation curves (**A**) were calculated for the controls (black) and hemophilia patients (red). The average prothrombin conversion curve at 1 pM TF were calculated for the same groups (**B**). Prothrombin conversion was quantified as PC_tot_ (**C**) and PC_max_ (**D**), and thrombin inactivation was measured by T-AT (**E**) and T-α_2_M (**F**). Reference ranges are depicted as grey boxes, dots show individual values and the lines indicate the median ± interquartile range. Statistical significance was tested by Mann–Whitney test and indicated as **** < p < 0.0001.

To illustrate the thrombin dynamics method further, we measured thrombin generation, prothrombin conversion and thrombin inactivation in 8 hemophilia A patients. Figure 6A,B show the average thrombin generation curve and prothrombin conversion curve at 1 pM TF in hemophilia A patients and 60 healthy male controls. Both the rate of prothrombin conversion and the amount of prothrombin converted is reduced in hemophilia (Fig. 6C,D). Subsequently. T-AT and T-α_2_M complex formation was significantly lower in patients compared to controls (Fig. 6E,F).

## Discussion

Thrombin is the central enzyme in coagulation by converting fibrinogen into fibrin thereby forming a clot. Any change in the coagulation cascade will be depicted in the generation and activity of thrombin. Thrombin generation is a sensitive method to investigate these changes but this method will not provide detailed reasons why the coagulation behaves differently in certain situations. In the current study, we hypothesized that each specific coagulation factor has a ‘fingerprint’, a specific influence on thrombin dynamics, which is initially revealed as a deviation in the TG profile. Thrombin dynamics is a method that goes further into detail quantifying the prothrombin conversion and the thrombin decay. By using thrombin dynamics we were able pinpoint how factor II, V, X, antithrombin and α_2_M affect the generation of thrombin.

Changes in thrombin generation are caused by specific changes in the underlying processes of prothrombin conversion and thrombin inactivation. Prothrombin conversion is not only influenced by procoagulant factors prothrombin and FX, but also by antithrombin. The total amount of prothrombin conversion is dependent on the available amount of prothrombin in the plasma, which is intuitively logical. In contrast, the maximum velocity of prothrombin conversion is not only dependent on the plasma level of prothrombin and FX, but also on the antithrombin level. This finding is in line with the data of Cvirn et al. that show that an increase of antithrombin levels reduces the amount of prothrombin fragment 1 + 2 formation^23^. Furthermore, according to the laws of enzyme kinetics, the prothrombin level is a rate limiting factor for the maximum prothrombin conversion velocity because it is the substrate for the prothrombinase complex^24^. The other (theoretical) rate limiting factor is the concentration of the enzyme of the prothrombinase complex, FX, which is confirmed by our experimental results. Therefore, a lower prothrombin or FX, and especially the combination of lower levels of both, result in a reduced PC_max_. Indeed, in a previous study we found a reduction of PC_max_ in patients on vitamin K antagonists^22^.

The formation of thrombin-antithrombin and thrombin-α_2_macroglobulin complexes depends on the plasma level of the respective inhibitor and the prothrombin level. Low prothrombin levels and subsequently low prothrombin conversion lead to low T-AT and T-αM formation, simply because thrombin needs to be available to be inhibited. Nevertheless, the antithrombin and α_2_Macroglobulin have a large effect on the amount of T-AT and T-α_2_M formed, respectively, and the ratio between the two types of enzyme-inhibitor complexes. In patients treated with heparin, whose anticoagulant effect is mediated through the facilitation of thrombin inhibition by AT^25^, the balance between T-AT and T-α_2_M shifts towards the formation of T-AT complexes. On the contrary, in subjects with low AT levels or high α_2_M levels, such as liver cirrhosis patients and young children, the balance between T-AT and T-α_2_M has been shown to shift in favor of T-α_2_M complexes^21,26^.

We have used thrombin dynamics analysis to study the process of prothrombin conversion and thrombin dynamics in multiple clinical settings. Thrombin generation shows a deviation in certain patients groups, but the deviation cannot be pinpointed to a specific target process in the coagulation cascade. We used thrombin dynamics analysis to provide more information of the mechanism of disease. Additionally, thrombin dynamics can be used to perform in silico experimentation, because of the computational model for thrombin inactivation that lies at its basis. This allows us to test research hypothesis in silico as illustrated below with several examples. To define what is abnormal we defined normal values for thrombin dynamics parameters PC_tot_, PC_max_,T-AT, T-α_2_M, and TDC and compared these results to previously acquired thrombin dynamics data. Supplementary Table 2 shows an overview of previous clinical thrombin dynamics studies and how the respective patient populations relate to the newly established normal ranges^14–22^.

Liver cirrhosis causes disturbed blood coagulation due to the reduced production of pro- and anticoagulant factors, such as prothrombin, FV, FX, and antithrombin^27–29^. As a result, cirrhosis patients suffer from both bleeding (eg bruising, ruptured esophageal varices) and thrombosis (eg deep venous thrombosis, pulmonary embolism)^30,31^. Nevertheless, routine clinical tests such as the prothrombin time indicate increased bleeding risk, whereas thrombin generation correlates better with the hemostatic situation in cirrhosis patients^27^. We found that prothrombin and antithrombin levels were significantly reduced to respectively 43% and 39% in severe liver cirrhosis^21^. In contrast, α_2_Macroglobulin was increased up to twofold resulting in decreased PC_tot_, T-AT and TDC values in thrombin dynamics analysis. In addition, T-α_2_M levels are elevated compared to healthy subjects and newly established reference values.

Children hardly ever suffer from thrombosis^32^ and thrombin generation is reduced in children compared to adults^33–35^. We recently investigated the mechanism behind this change and found that prothrombin conversion (PC_tot_ and PC_max_) is lower in children compared to adults^20^. Additionally, the balance between antithrombin and α_2_M for the inhibition of thrombin shifts more towards α_2_M. Nevertheless, liver disease in children causes striking similarities to the changes in adults^36^. However, the impressive reduction of prothrombin conversion provides an explanation for the diminished risk of pediatric thrombosis.

Hemophilia A is a genetic disorder that causes low or undetectable levels of the pro-coagulant FVIII and subsequently, patients have a severe risk of bleeding. Thrombin generation, prothrombin conversion and thrombin inactivation are reduced in hemophilia^6,37^, and has been used in the past to estimate a patients risk of bleeding^38^. A novel treatment approach in hemophilia is to attack the anticoagulant pathway, in an attempt to bring the pro- and anticoagulant process in balance^11^. We used the thrombin dynamics method and in silico simulations to investigate the effect of antithrombin-targeting in haemophilia A patients on thrombin dynamics and thrombin generation (unpublished work). A 50% reduction of antithrombin could increase thrombin generation peak height in haemophilia A patients by 80%. However, the variation in effect between patients was large, depending on their initial thrombin generation profile, and therefore, pre-therapeutic dose adjustment using the thrombin dynamics method might be interesting.

The thrombin generation method and subsequently the quantification of prothrombin conversion and thrombin inactivation have some limitations associated with in vitro testing. One limitation is the absence of platelets in the measurements in the current work. However, thrombin generation can also be measured in platelet rich plasma^39^. Furthermore, we have recently published that thrombin dynamics analysis can also be performed in platelet rich plasma and that the measurement parameters are sensitive to the platelet number^40^. Another limitation is the lack of the vessel wall, which plays a role in in vivo coagulation. In thrombin generation, this can be mimicked partially using soluble thrombomodulin^1^. Therefore, the results of thrombin generation, prothrombin conversion and thrombin inactivation measurements in vitro can be different than in vivo. Nevertheless, thrombin generation has been shown to be a useful indicator of bleeding and thrombosis risk^2–8^.

In conclusion, we show that prothrombin conversion is mainly influenced by prothrombin, FX and antithrombin levels, whereas thrombin inactivation is dependent on antithrombin and fibrinogen. Our study provides a better insight into the relation between coagulation factors and dynamic thrombin activity. The established reference values of thrombin dynamics will provide guidance values for clinically ‘normal’ and ‘abnormal’ thrombin dynamics parameter values. Our approach allows a more detailed insight into the mechanistic background of alterations of coagulation in specific patient populations and contributes in the design of therapeutic strategies in hemostatic diseases.

## Methods

### Sample collection

Our study protocols were evaluated by the local medical ethical boards (Medical Ethical Committee of Maastricht University Medical Center or Erasmus Medical Center Rotterdam). All research was performed in accordance with the relevant guidelines and regulations, and all volunteers gave full informed consent according to the Helsinki declaration. The study population consisted of 122 healthy adult individuals, aged 18–65 years. None of the participants used oral anticoagulant or anti-platelet drugs for at least two weeks, had a history of thrombosis or bleeding. Additionally, 8 haemophilia A patients were included. Blood was collected into vacuum tubes (1 volume trisodium citrate 0.105 M to 9 volumes blood) (BD Vacutainer System/Greiner). Platelet-poor plasma (PPP) was obtained by double centrifugation at 2630 g for 10 min and stored at − 80 °C until further use.

### Materials

Hepes buffers containing 5 mg/ml or 60 mg/ml bovine serum albumin were used to dilute the reagents or substrates, respectively, as described before^13^. Bovine serum albumin and unfractionated heparin and were purchased at Sigma-Aldrich (Darmstadt, Germany). The chromogenic thrombin substrate, S2238, was synthesized in house (Synapse Research Institute, Maastricht, the Netherlands)^41^. Bovine thrombin and bovine antithrombin were purified according to the protocols of Church et al. and Thaler et al. (Synapse Research Institute, Maastricht, the Netherlands)^42,43^.

### Coagulation factor determinations

All coagulation factor levels except α_2_-macroglobulin (fibrinogen, FII, FV, FX, and antithrombin) were determined on the STA-R Evolution analyzer (Diagnostica Stago, Asnières, France). Fibrinogen levels were measured with the Claus assay. Functional α_2_M levels were determined in house as previously described (Synapse Research Institute, Maastricht, the Netherlands)^14^.

### Thrombin generation

Calibrated Automated Thrombinography (CAT) was performed as previously described. PPP reagent low and PPP reagent, corresponding to 1 and 5 pM tissue factor (Diagnostica Stago, Asnières, France) were used according to the manufacturers description^13^. The results were analyzed with the Thrombinoscope software (Thrombinoscope, Maastricht, the Netherlands). The TG curves were used to perform additional computational analysis to extract prothrombin conversion curves^14^.

### Thrombin dynamics

The TG curve is the net result of prothrombin conversion and thrombin inactivation and therefore, the prothrombin conversion curve can be calculated from a TG curve.

Thrombin inactivation was predicted by the previously described and validated computational model^14,19,26,44^. This model consists of a set of ordinary differential equations, which describe the rate of thrombin inactivation in time based on the plasma AT, α_2_M and fibrinogen level and the free thrombin concentration at each point in time (Eqs. 1–3).

1 $${\text{d}}\left( {{\text{T}} - {\text{AT}}} \right)/{\text{dt }} = {\text{ k}}_{{{\text{AT}}}} \cdot \, \left[ {{\text{AT}}} \right]_{{\text{t}}} \cdot \, \left[ {{\text{T}}_{{{\text{free}}}} } \right]_{{\text{t}}}$$

2 $${\text{d}}({\text{T}} - \alpha_{{2}} {\text{M}})/{\text{dt }} = {\text{ k}}_{{\alpha {\text{2M}}}} \cdot \, [\alpha_{{2}} {\text{M}}\left] {_{{\text{t}}} \cdot \, } \right[{\text{T}}_{{{\text{free}}}} ]_{{\text{t}}}$$

3 $$- {\text{d}}\left( {{\text{T}}_{{{\text{free}}}} } \right)/{\text{dt }} = {\text{k}}_{{{\text{AT}}}} \cdot \, \left[ {{\text{AT}}} \right]_{{\text{t}}} \cdot \, \left[ {{\text{T}}_{{{\text{free}}}} } \right]_{{\text{t}}} + {\text{ k}}_{{\alpha {\text{2M}}}} \cdot \, [\alpha_{{2}} {\text{M}}\left] {_{{\text{t}}} \cdot \, } \right[{\text{T}}_{{{\text{free}}}} ]_{{\text{t}}}$$

The amount of thrombin that is free in solution (T_free_) depends on the amount of thrombin substrate that is present, and rate constants for the inactivation of thrombin by antithrombin (k_AT_) and α_2_-macroglobulin (k_αM_) are dependent on the plasma fibrinogen level, as described in more detail elsewhere^14^.

At any moment during the course of the TG process, the TG curve is the net result of prothrombin conversion and thrombin inactivation. Therefore, the course of prothrombin conversion (d(P)/dt) can be calculated from the TG curve ([T]t) and the inactivation rate of thrombin at a specific thrombin concentration (d(T-inh)/dt) (Eq. 4). With the previously described model for thrombin inactivation we can calculate the thrombin inactivation rate at each time point during TG (Eq. 5).

4 $${\text{d}}\left( {\text{T}} \right)/{\text{dt }} = \, - {\text{d}}\left( {\text{P}} \right)/{\text{dt }} - {\text{ d}}\left( {{\text{T}} - {\text{inh}}} \right)/{\text{dt}}$$

5 $$- {\text{d}}\left( {\text{P}} \right)/{\text{dt }} = {\text{ d}}\left( {\text{T}} \right)/{\text{dt }} + {\text{ k}}_{{{\text{AT}}}} \cdot \, \left[ {{\text{AT}}} \right]_{{\text{t}}} \cdot \, \left[ {\text{T}} \right]_{{\text{t}}} + {\text{ k}}_{{\alpha {\text{2M}}}} \cdot \, [\alpha_{{2}} {\text{M}}\left] {_{{\text{t}}} \cdot \, } \right[{\text{T}}]_{{\text{t}}}$$

Thrombin inactivation can be determined independently of the thrombin generation curve as a parameter called the thrombin decay capacity. This is the pseudo-first order decay constant for thrombin that combines the overall effect of thrombin inactivation by AT and α_2_M. In the thrombin dynamics method, the prothrombin conversion curve is quantified by its area-under-the-curve, which is the total amount of prothrombin converted (PC_tot_) throughout the TG experiment, and the peak height of the prothrombin conversion curve, which is the maximum rate of prothrombin conversion (PC_max_).

### Statistical analysis

Statistical analysis was performed using Graphpad Prism (version 8, San Diego, USA). Reference ranges were determined as the 2.5th and 97.5th percentile values of the healthy subjects dataset variables. Data was presented as the median ± interquartile range. Statistical significance was determined by ANOVA analysis with Bonferroni correction or the Mann–Whitney test, dependent on the number of groups that needed to be analyzed. Dose–response effects were investigated using linear correlation. Correlations were calculated as the Pearson correlation coefficient in the healthy subject sample data. A p-value below 0.05 was considered statically significant.

## Supplementary information

Supplementary file 1
