# Supplementary material for: Deciphering the coagulation profile through the dynamics of thrombin activity
Source: Sci Rep. 2020 Jul 27;10:12544. doi: 10.1038/s41598-020-69415-y (PMC7385119; doi:10.1038/s41598-020-69415-y)
Supplement: Supplementary file 1 [file 41598_2020_69415_MOESM1_ESM.docx]

**Supplementary material**

Deciphering the coagulation profile through the dynamics of thrombin activity

**Running title:** The dynamics of thrombin generation

Romy M.W. de Laat – Kremers^1,2*^, Qiuting Yan^1,2^, Marisa Ninivaggi^1^, Moniek de Maat^3^, B. de Laat^1,2^

^1^Synapse Research Institute, Maastricht, the Netherlands

^2^Dept of Biochemistry, CARIM, Maastricht University, Maastricht, the Netherlands

^3^Dept of Hematology, Erasmus University Medical Center, Rotterdam, the Netherlands

**Keywords:** thrombin dynamics, thrombin generation, thrombin inactivation, prothrombin conversion, coagulation factors

***Corresponding author:**

Name: Romy de Laat - Kremers

Address: Pastoor Habetsstraat 50, 6217KM, Maastricht, the Netherlands

Email: r.delaat@thrombin.com

Phone: +31 681032543

**Supplementary figure 1: The effect of prothrombin and antithrombin on thrombin generation.** **(A-F)** Prothrombin deficient plasma was mixed with pooled normal to achieve plasma concentrations of 0, 5, 10, 20, 30, 40, 50, 60, 70, 80, 90, and 100% prothrombin. Thrombin generation curves at 1 (A) and 5 pM TF (B) are shown (0-100% prothrombin from bottom to top) and thrombin generation parameters lag time (C), the peak (D), time-to-peak (E) and ETP (F) were quantified at 1 pM TF (■ symbols) and 5 pM TF (● symbols). **(G-L)** Antithrombin deficient plasma was mixed with pooled normal to achieve plasma concentrations of 40, 50, 60, 70, 80, 90, and 100% antithrombin. Thrombin generation curves at 1 (G) and 5 pM TF (H) are shown (0-100% antithrombin from top to bottom) and thrombin generation parameters lag time (I), the peak (J), time-to-peak (K) and ETP (L) were quantified at 1 pM TF (■ symbols) and 5 pM TF (● symbols). The average results of 3 experiments are shown as the mean ± SD.

**Supplementary figure 2: The effect of coagulation factors on the thrombin decay capacity.** The dose-response relationships of the thrombin decay capacity and the plasma prothrombin (A), FV (B), FX (C) and antithrombin level (D).

**Supplementary figure 3: The effect of FV and FX on thrombin generation.** **(A-F)** FV deficient plasma was mixed with pooled normal to achieve plasma concentrations of 0, 5, 10, 20, 30, 40, 50, 60, 70, 80, 90, and 100% FV. Thrombin generation curves at 1 (A) and 5 pM TF (B) are shown (0-100% FV from bottom to top) and thrombin generation parameters lag time (C), the peak (D), time-to-peak (E) and ETP (F) were quantified at 1 pM TF (■ symbols) and 5 pM TF (● symbols). **(G-L)** FX deficient plasma was mixed with pooled normal to achieve plasma concentrations of 0, 5, 10, 20, 30, 40, 50, 60, 70, 80, 90, and 100% FX. Thrombin generation curves at 1 (G) and 5 pM TF (H) are shown (0-100% FX from bottom to top and thrombin generation parameters lag time (I), the peak (J), time-to-peak (K) and ETP (L) were quantified at 1 pM TF (■ symbols) and 5 pM TF (● symbols). The average results of 3 experiments are shown as the mean ± SD.

**Supplementary table 1: Reference values of thrombin dynamics parameters.**

|  | **Parameter** | **Median** | **2.5^th^ percentile** | **97.5^th^ percentile** |
| --- | --- | --- | --- | --- |
| **1 pM TF** | PC_tot_ (nM) | 1036 | 693 | 1344 |
|  | PC_max_ (nM) | 213 | 109 | 415 |
|  | T-AT (nM) | 1001 | 667 | 1283 |
|  | T-α_2_M (nM) | 33 | 16 | 63 |
|  |  |  |  |  |
| **5 pM TF** | PC_tot_ (nM) | 1057 | 746 | 1335 |
|  | PC_max_ (nM) | 250 | 153 | 474 |
|  | T-AT (nM) | 1021 | 729 | 1279 |
|  | T-α_2_M (nM) | 34 | 16 | 63 |
|  |  |  |  |  |
| **Independent of TF concentration** | TDC (min^-1^) | 0.816 | 0.635 | 1.002 |

**Supplementary table 2: Comparison of previous thrombin dynamics data with the current reference values^14,17,18,20-22,39^.**

| **Journal, Year** | **Patient population** | **PCtot**  **(746-1335 nM)** | **PCmax**  **(153-474 nM/min)** | **TAT**  **(729-1279 nM)** | **Ta2M**  **(16-63 nM)** | **TDC**  **(0.633 – 1.002 min^-1^)** |
| --- | --- | --- | --- | --- | --- | --- |
| J Thromb Haemost. , 2015 | Liver cirrhosis | Reduced | Within range | Reduced | Within range | Reduced |
|  | Kidney failure | Reduced | Within range | Within range | Within range | Reduced |
| Thromb Haemost., 2016 | Pediatrics | Reduced or within range | Reduced or within range | Reduced or within range | Increased or within range | Reduced or within range |
| Thromb Haemost., 2016 | After strenuous exercise | Within range | Increased or within range | Within range | Within range | Within range |
| Thromb Haemost., 2016 | After surgery with cardiopulmonary bypass | Reduced | Reduced or within range | Reduced | Within range or increased | Reduced |
| PLoS One., 2017 | Severe liver cirrhosis | Reduced | Within range | Reduced | Increased in part of the patients | Reduced |
| Scientific Reports, 2017 | Asthma patients | Within range | Data not shown | Within range | Within range | Data not shown |
| Blood Adv., 2018 | Antiphospholipid syndrome patients | Within range or increased | Within range or increased | Data not shown | Data not shown | Within range |
| Thromb. Res., 2020 | Severe prediatric liver disease | Reduced | Within range | Reduced | Within range or increased | Reduced or within range |

Thrombin generation data obtained at 5 pM TF was analyzed; if a parameter value was outside of the normal range for a part of the patient population, this is noted in the table as either reduced or increased.
